# Utility-scale solar PV performance enhancements through system-level modifications

**DOI:** 10.1038/s41598-020-66347-5

**Published:** 2020-06-29

**Authors:** Andrew Glick, Naseem Ali, Juliaan Bossuyt, Marc Calaf, Raúl Bayoán Cal

**Affiliations:** 1 Department of Mechanical and Materials Engineering, Portland State University, Portland, OR 97207 USA; 2 Department of Mechanical Engineering, University of Utah, Utah, UT 84112 USA

**Keywords:** Solar energy, Fluid dynamics

## Abstract

Performance of solar PV diminishes with the increase in temperature of the solar modules. Therefore, to further facilitate the reduction in cost of photovoltaic energy, new approaches to limit module temperature increase in natural ambient conditions should be explored. Thus far only approaches based at the individual panel level have been investigated, while the more complex, systems approach remains unexplored. Here, we perform the first wind tunnel scaled solar farm experiments to investigate the potential for temperature reduction through system-level flow enhancement. The percentage of solar irradiance converted into electric power depends upon module efficiency, typically less than 20%. The remaining 80% of solar irradiance is converted into heat, and thus improved heat removal becomes an important factor in increasing performance. Here, We investigate the impact of module inclination on system-level flow and the convective heat transfer coefficient. Results indicate that significant changes in the convective heat transfer coefficient are possible, based on wind direction, wind speed, and module inclination. We show that 30–45% increases in convection are possible through an array-flow informed approach to layout design, leading to a potential overall power increase of ~5% and decrease of solar panel degradation by +0.3%/year. The proposed method promises to augment performance without abandoning current PV panel designs, allowing for practical adoption into the existing industry. Previous models demonstrating the sensitivity to convection are validated through the wind tunnel results, and a new conceptual framework is provided that can lead to new means of solar PV array optimization.

## Introduction

The operating temperature has a significant effect on the cost of photovoltaic (PV) solar energy. PV panels in the field often operate 20–40 °C above their rated temperatures, and each rising degree decreases both panel efficiency and lifetime^1–3^. For example, in a typical utility scale PV installation in Colorado, summer ambient temperatures average 28.6 °C and the panel nominal cell operating temperature (NOCT) averages 48.2 °C with summer maximum module temperatures reaching 59 °C. This increase becomes important as a 5 °C increase in temperature with respect to the standard test condition (STC) has the effect of decreasing the panel efficiency 1–3%^4,5^. Therefore, these sizeable effects make temperature reduction a key strategy on the roadmap to lowering solar energy costs^6^. Two general strategies exist to try to achieve this goal. The first is to maximize cooling through enhanced convection/conduction and radiative cooling, and the second minimizes the thermal load through increased efficiency or advanced reflectance^7^. A variety of techniques have been proposed to lower panel temperature for individual panels including phasechange materials, heat sinks, and active methods such as air and water cooling^8^. For example, Krauter^9^ used spray water (4.4 *L*/*min* *m*^2^) to increase the performance of the M55 module by 1.5%. Abdolzadeh and Ameri^10^ improved the performance by 1.8%. Odeh and Behnia^11^ reduced the operating temperature and electrical yield increased in the range of 4–10% by spraying 4 *L*/*min*. Hosseini *et al*.^12^ showed an increase in the efficiency of PV modules of 60 W by 3.66% with spraying water. However these methods have not proved to be commercially viable due to their extra cost and/or maintenance requirements^13,14^. Within the first strategy, no work has been performed to investigate means to enhance convective cooling of solar modules at the array-level^13–16^, despite the potential gains of this approach^7^.

In existing full scale solar arrays, varying system-level parameters such as row spacing, inclination angle, height from ground, and row orientation relative to predominant wind direction is impractical. Up until now, no scaled platform has existed for studying enhancements at the array level. This lack thereof has meant that any study on the array level has been limited to sensitivities of the convective heat transfer on wind speed^17,18^. The experimental platform introduced in this work provides for the first time the opportunity to shift the focus from the individual panel to the array. Through this new lens, we examine the fluid flow and heat transfer in a large scaled solar array, beginning with fundamental parameters of inclination angle and wind speed. Flow passing over and through a solar array can interact with the panels in distinctly different manners depending on these two parameters. Certain inclination angles can cause more turbulent mixing, increasing convection heat transfer while others redirect the flow in directions that can enhance or reduce convection. Here, we seek to develop an understanding of the fluid mechanisms that drive the heat transfer, and determine what magnitude of temperature reductions are possible through enhanced array convection. Further, the present study provides an opportunity for future work by examining the key parameters that govern heat transfer in large solar farms and ultimately informing improved layout designs^19^. This approach to temperature reduction is particularly attractive as it is passive, and does not require costly new technology developments or maintenance. Thus, large solar farms with enhanced convection have the potential to have temperature reductions whilst still providing a similar aesthetic and utilizing existing labor skillsets to install and maintain.

Our experimental results show that the sensitivity to wind direction and module inclination angle is significant, with a 45% increase in convective heat transfer coefficient possible, depending on the incoming wind direction. These changes can be directly correlated to changes in the flow field through the farm. With current solar farm installations constructed agnostic to site predominant wind direction, this suggests that room may exist for site-specific row and spacing design. This is not to suggest that the inclination angle should be altered or optimized for convection enhancement. However, this research does suggest the importance of predominant wind direction and sub-panel flow, and this may be able to be taken advantage of in a variety of ways. One example in which a farm may be able to optimize for this in the design stage would be to use a larger E-W direction compared to the N-S direction in the case that the predominant wind comes from the North or South. This plant uses the same area, but has more panels exposed to faster moving inflow and thus improved cooling. For sites with low wind speeds over much of the year, this research suggests further examination to determine if the buoyant currents generated by the heated air above the panels themselves could be harnessed to increased mixing and flow through the solar farm.

## Solar Array Experimental Platform

**Figure 1:**
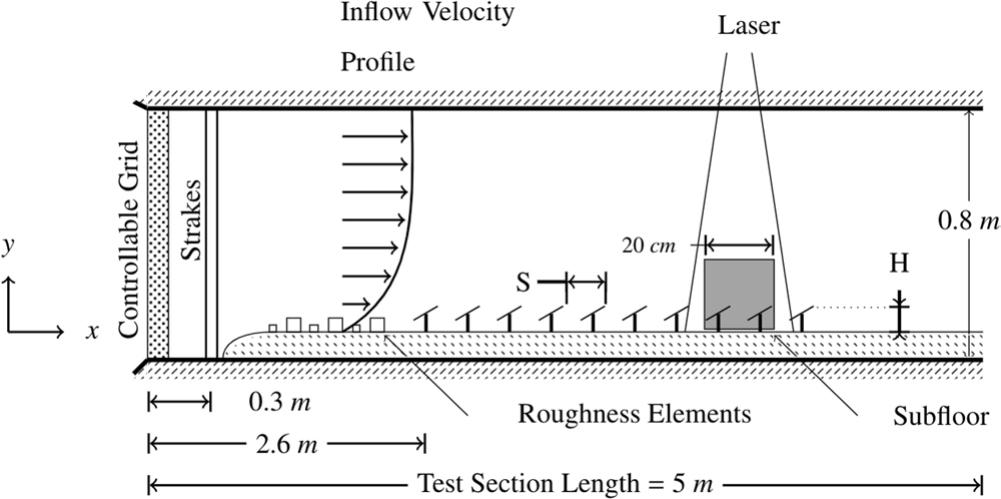
Schematic of wind tunnel experimental setup, side view. Flow is from left to right. Roughness elements shown as squares represent chains of two distinct sizes. Panels shown at angle of +30°. Greyed square represents where images were captured using the PIV camera. Schematic not to scale.

To be able to explore optimizations in the system-level parameter space, a new experimental platform was required. This solution had to scale properly to utility-scale, have sufficient rows to ensure flow convergence, and be highly configurable to a variety of angles, spacings, ground-mount heights, and wind directions^20^. The resulting platform is shown in Fig. 1, where 40 individual panels are arranged into 10 rows. Key to satisfying scalability is the recreation of an incoming flow mimicking the atmospheric boundary layer, achieved with tapered blockage elements (strakes) followed by surface roughness elements (chains). Flow field measurements with high-spatial and temporal resolution were achieved with particle image velocimetry (PIV). This experimental platform allowed us to explore a question that was previously impractical to answer at full scale and identify the highest reward paths to PV farm layout optimization for lower operating temperatures.

In addition to mimicking the atmospheric boundary layer, further steps were taken to validate the applicability of the experimental setup to utility scale solar farms. Convergence tests were completed to analyze the flow and convective heat transfer coefficient to ensure that enough rows were present to create a fully developed flow. It was found that the flow became fully developed after 6–7 rows, and thus 10 rows were used, and data taken between the 8th and 9th rows. Scalability of the experiment was determined through experiments examining flow structure changes with inflow wind speed/Reynolds Number. Reynolds number independence was observed. Demonstrating this is critical to establish scalability, since a comparison of the Reynolds number between a full scale farm and the experimental setup is approximately equal to the size ratio of the panels (full scale to scaled panels). Hence, dynamic similarity on the individual panel level will certainly not be achieved. However, the focus of this study is on the large-scale transport properties of the turbulence, where Reynolds number effects are known to be less dominant.

**Figure 2:**
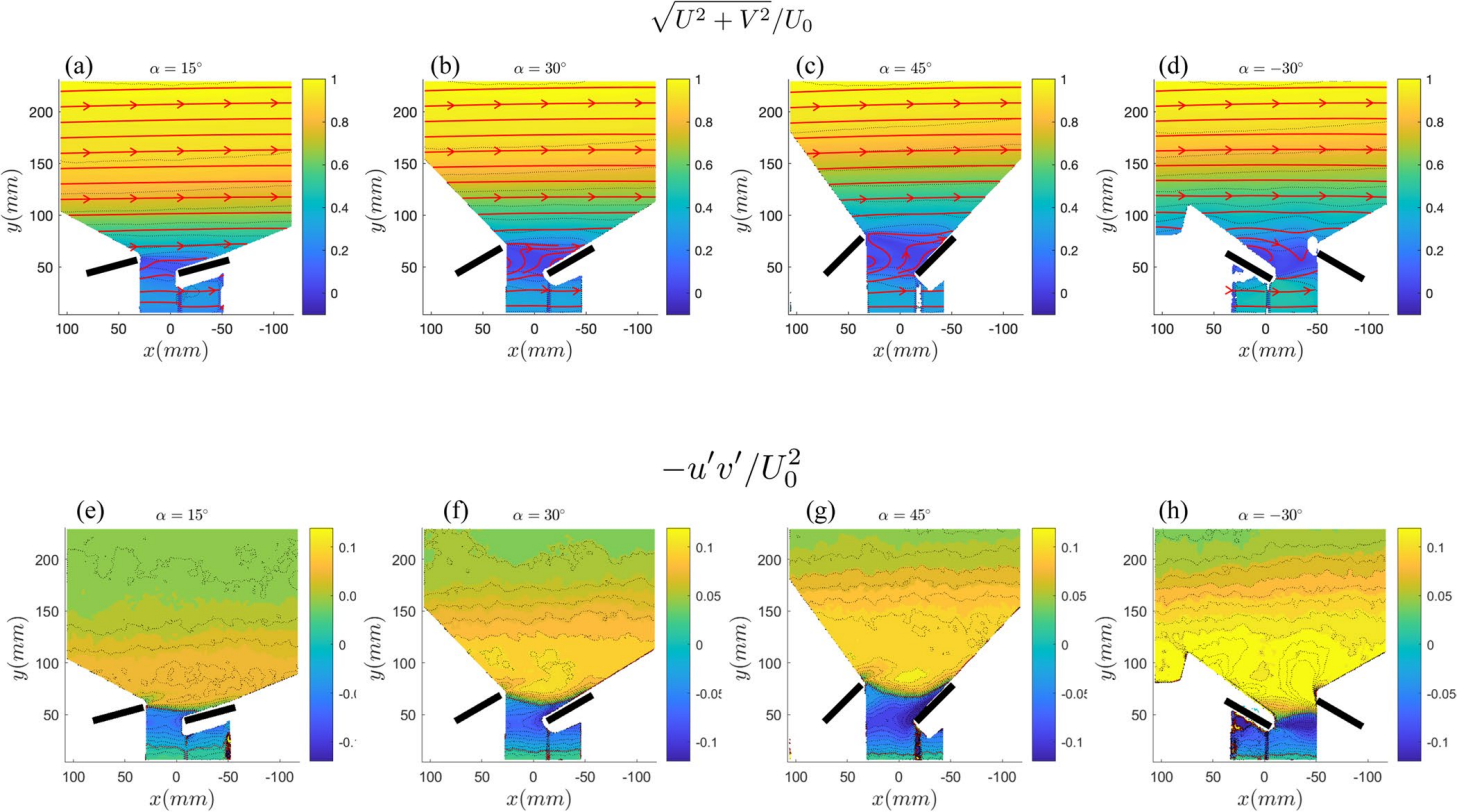
Normalized mean velocity and Reynolds shear stress profiles across inclination angles plotted in the PIV spatial window. Data is taken between the 8^*th*^ and 9^*th*^ rows in the solar farm where a convergence study demonstrated fully developed flow. Flow is from left to right. Red lines indicate the boundary between positive and negative values.Black lines represent the locations of the physical panels, which obstruct the view of the camera in the corners of the measurement window (obstruction shown as white) and prevent data from being taken. Results shown here were taken at nominal wind speed and power input values of 3.9 *ms*^−1^ and 450 *Wm*^−2^. (**a**) $\sqrt{{U}^{2}+{V}^{2}}/{U}_{0}$ at $\alpha =15^\circ$, (**b**) $\sqrt{{U}^{2}+{V}^{2}}/{U}_{0}$ at $\alpha =30^\circ$, (**c**) $\sqrt{{U}^{2}+{V}^{2}}/{U}_{0}$ at $\alpha =45^\circ$, (**d**) $\sqrt{{U}^{2}+{V}^{2}}/{U}_{0}$ at $\alpha =-\,30^\circ$, (**e**) $-\langle {u}^{{\prime} }{v}^{{\prime} }\rangle /{U}_{0}^{2}$ at $\alpha =15^\circ$, (**f**) $-\langle {u}^{{\prime} }{v}^{{\prime} }\rangle /{U}_{0}^{2}$ at $\alpha =30^\circ$, (**g**) $-\langle {u}^{{\prime} }{v}^{{\prime} }\rangle /{U}_{0}^{2}$ at $\alpha =45^\circ$ and (**h**) $-\langle {u}^{{\prime} }{v}^{{\prime} }\rangle /{U}_{0}^{2}$ at $\alpha =-\,30^\circ$.

Using the solar array experimental platform presented above, we performed a series of experiments to determine the relationship between the panel inclination angles, inflow wind speed and convection heat transfer coefficient. Four inclination angles were chosen with respect to the main streamwise direction, representative of standard arrangements at different latitudes: [15°, 30°, 45°, −30°]. The negative inclination angle represents the same standard solar farm with 30° inclination for which the wind inflow is perpendicular to the back side of the panels. Figure 2 illustrates the flow field (normalized, ensemble-averaged velocity magnitude spatial contours and Reynolds shear stress) for the four inclination angles.

## Characterization of momentum and heat transfer

Normalized velocity magnitude $\sqrt{{U}^{2}+{V}^{2}}/{U}_{0}$, or the resultant velocity vector normalized by the freestream velocity, is compared for the different cases beginning with top row of Fig. 2. U and V refer to the streamwise (horizontal) and wall-normal (vertical) directions, and u’ and v’ refer to the fluctuations about the mean in these directions. As expected, a well defined wake region is observed directly behind the panels for all angles. As indicated by the flow vectors, even a flow reversal can take place in several cases directly behind the panels. On this regard, it is of interest to realize that the spatial area with recirculating flow behind the panel increases with inclination angle, indicating an enhanced velocity deficit. Further, there is a reduction on mean advection (or transport of momentum by the bulk fluid motion) at all heights above the panel as the angle increases from 15° to 45°.

Comparing two of these configurations, the +30° case (Fig. 2a) with the −30° case (Fig. 2d), significant variations are observed in the sub-panel region. In comparison with the +30° case, the −30° case exhibits sub-panel velocities higher by almost 40%. This has great importance to the resulting heat transfer, as the extent of the cooling that can be accomplished with any given array configuration depends on the way in which incoming flow is divided into array flow and bypass flow. We refer to array flow as that flow traversing underneath and between the panels, while we refer to bypass flow as the incoming flow that does not or only slightly interacts with the array. When angled at −30°, the panels effectively act as downward baffles, guiding flow that would have been deflected upwards and channeling it underneath the panels, as demonstrated in the streamlines in Fig. 2a through 2d. The net effect is that an increased volume of fluid interacts with the panels compared with the +30° case (i.e. an increase in array flow relative to bypass flow). Further, the increase in streamwise subpanel flow shown in Fig. 2d is explained in part by the change in the Reynolds shear stress shown in Fig. 2h. The magnitude of the Reynolds shear stress is an indication of the turbulent mixing in the flow, and the sign of the shear stress indicates whether the flow is being entrained upwards (+) or downwards (−). The quantity is normalized by the square of the freestreem velocity. Increased values of $-\langle {u}^{{\prime} }{v}^{{\prime} }\rangle /{U}_{0}^{2}$ above the panel are directly proportional to increases in the vertical transport of momentum, which is an effective mechanism for replenishing the mean kinetic energy throughout the panel array, including the region below the panels. Further, this enhanced vertical momentum exchange facilitates a steady supply of cooler air from aloft to interact with the solar modules.

**Figure 3:**
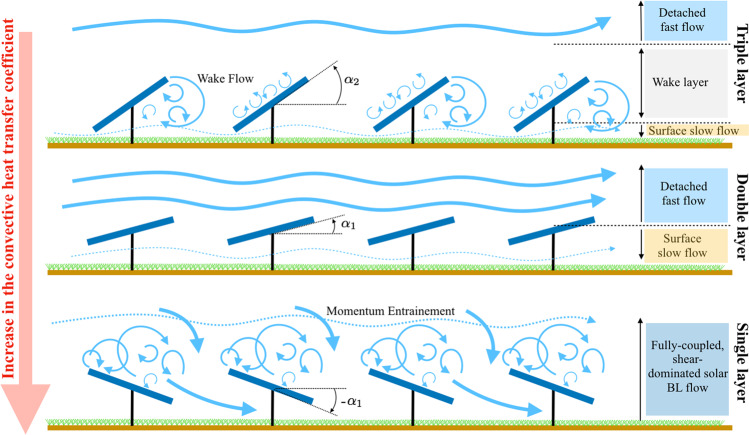
Effect of solar farm arrangement on flow: (Level 1, Triple layer) Higher inclination angles, larger velocity deficits, high turbulent mixing, little sub-panel flow (Level 2, Double layer) Low inclination angles, low interaction with overhead flow, low mixing (Level 3, Single layer) increased shear leading to increased sub-panel flow.

Therefore, based on the results from Fig. 2, we introduce a conceptual connection between the convective heat transfer coefficient and the solar farm turbulent flow structure. This is schematically represented in Fig. 3. Increasing heat transfer through arrays of heated elements depends largely on two properties - the bulk mean velocity through the panels and the turbulent mixing. Concerning the first, bulk advection is largely a function of the local wind speed over the upper and lower surfaces of the solar farm, and can be augmented through spacings that prevent dead-spots and encourage key flow channels. Turbulent mixing on the other hand increases interaction with the overhead flow, and causes the turbulent boundary layer to grow more quickly over the surface of the panels and have larger convection coefficients due to the enhanced mixing of air masses with different temperatures. Figure 3 illustrates these two concepts and their effect on overall heat transfer. Three forms of interaction characterize how solar farms interact with the incoming flow. In the triple layer flow shown at the top of Fig. 3, a steep panel inclination angle causes two competing effects; large eddies are shed from the edges of the panels, increasing turbulent mixing while increased area normal to the incoming flow creates a larger velocity deficit. For the double layer flow, minimal interaction is fostered between the panels and the overhead flow, as no significant turbulence is induced. For both the triple and double layer flows, there is limited encouragement for the flow to pass underneath the panels. This is contrasted with the single layer flow shown at the bottom of Fig. 3, which is applicable to the case where the flow is coming at the farm from the rear. In this case, the panels act as downward baffles, increasing the Reynolds shear stress above the panels which in turn pulls the flow that would have been deflected upwards for the positive inclination angle cases into interaction with the panels.

**Figure 4:**
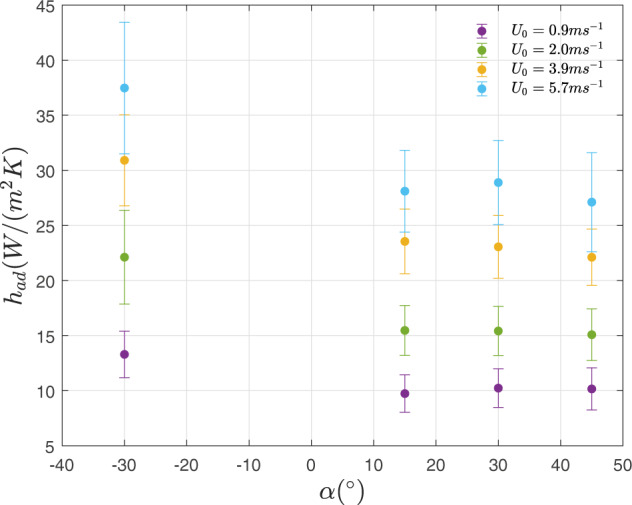
Experimental results of panel inclination angle vs Adiabatic heat transfer coefficient number for a panel in the 8th row of the model solar farm. All *h*_*ad*_ shown are averaged between front and rear surfaces. Tests were completed at four wind tunnel test section speeds ranging from 0.9 *ms*^−1^ to 5.7 *ms*^−1^.

**Figure 5:**
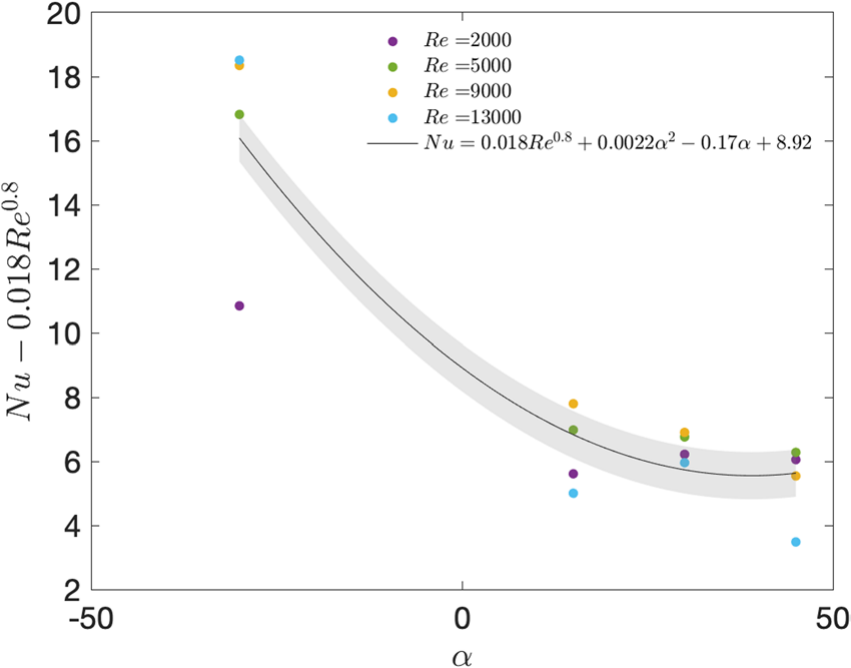
Experimental results of panel inclination angle vs Reynolds adjusted Nusselt number for a panel in the 8th row of the model solar farm.

The increase in convection heat transfer shown on the left in Fig. 3 is demonstrated in the measured values of convective heat transfer coefficient shown in Fig. 4. We first characterized the averaged adiabatic convective heat transfer coefficient across four velocities and four panel inclination angles (Fig. 4). The upper and lower surface *h*_*ad*_ values were averaged. When averaged between upper and lower surfaces, the contrast between the −30° and +30° cases stands out in Fig. 4, where increases between 30% to 45% were observed across the range of velocities measured. Between the positive angle cases, trends are more difficult to ascertain given the measurement uncertainty, however a slight upward trend is observed and can be further noted in Fig. 5.

In engineering, heat transfer relations are traditionally expressed as correlations between the Reynolds number and the Nusselt number. Reynolds number represents the ratio of inertial to viscous forces and the Nusselt number the ratio of conduction to convection heat transfer. Given the magnitude of variation observed between the different angles, this study suggests that the panel inclination angle should be added as a parameter in these equations. A correlation is proposed and is shown in Fig. 5. Although the variation between the positive angles tested was within the measurement uncertainty and more angles should be tested between −45° and +15°, it is evident that the varied flow field caused by a change in inclination angle should be factored into future solar farm correlations. It is not expected that this trend would continue upwards as angle decreases to −45° and above, but indicates that there is an optimum negative angle where maximum heat transfer can be achieved. These results have significant implications for those designing a solar farm, for example, at a site located in the northern hemisphere with year-round or summer daytime prevailing wind direction from the north. In this case, this work allows designers to begin to further optimize farm inclination angle and calculate the tradeoffs between the lower temperature, incident radiation, shading, and more.

## Theoretical enhancement due to convection

**Figure 6:**
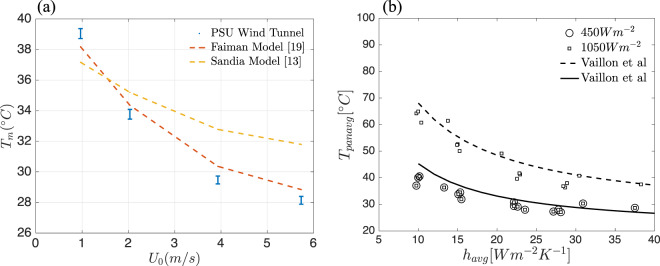
Models showing the temperature dependence on convection heat transfer coefficient *h* and wind speed *U*. (**a**) Wind speed effect on module temperature and (**b**) Convection heat transfer coefficient effect on module temperature at two different irradiance values.

Although no other existing models parameterize inclination angle in utility-scale PV farms, our results are consistent with the current literature across the Reynolds numbers tested, and demonstrate the ability of the scaled solar farm to represent full scale PV farms. Fig. 6a shows the experimental results from the scaled model farm compared with the Faiman model, which is extensively used in thermal PV models^21^. The notable drops in temperature observed between 1–2 *ms*^−1^ take on more significance when viewed alongside the understanding that wind speed on the edge of a solar farm may be significantly reduced as one proceeds inwards due to wake and blockage effects. Hence, if array setups could be organized such to eliminate or reduce the wake effect such that a higher wind speed is observed further into the solar farm, Fig. 6a shows that temperature reductions of 4–6° could be possible.

Further, to understand how convection enhancement would effect module temperature, relationships between the convection heat transfer coefficient *h*_*ad*_ and the module temperature were compared between theory and the experiments. Fig. 6b shows the theoretical and empirical improvements to the panel operating temperature resulting from improvements to the panel average convective heat transfer coefficient. A simplified energy balance model developed by Vallion *et al*. to investigate the pathways for enhancing solar PV efficiency, with some of the results are shown as solid lines^7^. Data points represent mean temperatures for cases measured in this experiment. Theory and the experimental results agree well, as indicated by the represented results. Theory predicts that the maximum impacts to temperature should be observed when increasing *h*_*ad*_ near its lowest values. Our experiment confirms this, where an increase in *h*_*ad*_ from 10 to 15 *Wm*^−2^ *K*^−1^ at constant inclination angle of +30° decreases the temperature from 60.6 °C to 50.1 °C. If sustained, a 10 °C decrease in temperature implies a 3–5% increase in efficiency. Following Jones-Albertus *et al*.^22^, the impact of changes in system parameters on the levelized cost of energy were examined. The current approach decreases the degradation rate by 0.3%/year^23^. The potential for boosting the reliability of solar PV system is achieved through lowering degradation rates, and consequently increasing durability and system lifetimes.

While the reduction in panel temperature and thus effect on annualized energy due to convection in a PV device is governed by seasonally dependent site-specific conditions such as ambient temperature, wind speed, and irradiance, the models presented in Fig. 6 show that the most significant impact on temperature is possible at the large wind speed or convective heat transfer. As many solar farms are close to the ground or not built at sites with high average wind speed, this indicates that small changes of heat transfer coefficient have potential for important changes in temperature of panels.

## Conclusion

Although progress is being made on increasing solar PV efficiency and maximizing power produced, challenges remain in decreasing PV panel operating temperatures. This study experimentally demonstrates the achievable enhancements in solar PV efficiency if PV arrays are designed to take advantage of convective cooling. A 30–45% increase in convective heat transfer coefficient was observed when the incoming flow direction shifts 180° to face the rear surface of the PV panels. This increase corresponds to a 5–9 °C decrease in PV module temperature. While changing the inclination angle of solar panels to optimize for convective cooling may be impractical or undesirable, this parametric study highlights the significant impact wakes, turbulence and sub-panel velocity have on panel operating conditions, through altering the convective heat transfer.

The current approach can be considered as a passive cooling mechanism that leads to the increase of conversion efficiency and reduces the irreversible damage to the PV-cell materials. The increase in connectivity reduces the reduction in open circuit voltage, fill factor and power output for PV cells. Compared to past studies that used active cooling systems, the current approach is considered as a promising alternative for the solar energy community. Following solar industry rules, solar panels last about 25–30 years, and degradation rates below 1% are common throughout the industry. With our approach, the degradation will be below 0.7% per year meaning that the solar panel will still be operating at approximately 85% of their efficiency.

The importance of these factors opens a pathway for future work to examine other parameters that may be easily optimized, such as panel row spacing, height of panel from the ground, and other structures or layouts that enhance flow channeling through the farm. In related applications, roof mounted arrays are often more adversely impacted by temperature, so questions arise around the possibilities of convection enhancement through parameters such as roof offset height. Further, this study stresses the importance of wind direction on PV energy yield, and provides values to be used in solar farm modeling systems that will lead to improvement of the existing models.

The convection enhancement strategies discussed herein began the conversation in the forced convection regime. However, it is important that future studies examine enhancements that may be possible using only natural convection since many solar sites are without large incoming velocities for much of the hotter months. Although this study examined forced convection relevant to higher wind sites, the same methodology could be applied in the regime of natural convection, and Fig. 6 demonstrates that small changes at the low end of inflow speeds could offer substantial temperature benefits. Further, any high-potential layout changes will have to be weighed against added material, land, or installation costs and future work should examine the tradeoffs therein.

## Methods

### Panel fabrication

A solar farm was experimentally simulated using forty model panels. Each panel is comprised of four layers, each 254 *mm* wide × 50.8 *mm* long: 6061 aluminum flat bar (1.6 *mm* thick), Kapton heater (0.1 *mm* thick), aerogel insulation (2 *mm* thick), and 6061 aluminum flat bar (1.6 *mm* thick). The complete panel structure had a thickness of 5.3 *mm*. Comparing with a full size solar farm with representative dimensions of 1.68 *m* long × 0.03 *m* thick placed side by side in long rows, these dimensions represent a 1:33 scale. Each panel was uniformly heated using a commercially available electric resistance heater, a Watlow 120 *Watt* 120 *Volt* Kapton heater. Electric power was supplied to the panels by two 48 *VDC* 6 *A* power supplies, with each power supply separately supplying twenty of the panels with power in parallel. The voltage drop across each panel measured with a digital multimeter on the Hewlett Packard (HP) data system described below. The voltage supplied to the heaters was maintained to within 19 *mV*.

### Temperature measurement

To measure the surface temperatures, Type T Copper constantan (±0.5 °C) thermocouples were placed underneath the top surface in a cavity 0.79 *mm*, 6.4 *mm* deep, where the center of the cavity was located 0.635 *mm* from the panel surface. Each cavity was filled with a thermally conductive silicone (Halnziue silicone heatsink plaster, HY910) and a thermocouple inserted to full depth. The thermocouple junction itself was kept to a length of 2 *mm* or less. All thermocouple lead wires were routed from their starting point along the edge of the module and covered with an anti-reflective gaffer tape. All temperature measurements were taken using Type T Copper Constantan (±0.5 °C) thermocouples (OMEGA TT-T-30-SLE), and recorded using an HP34970A data acquisition unit. Data was recorded at a frequency of 0.2 *Hz*, and averaged over 5 minute periods after the change in surface temperature varied less than 0.08 °C over a 5 *min* period. A single ice-point reference was used for the thermocouples. Each thermocouple was routed through a zone box to ensure reference junction temperature uniformity. Ten out of the forty panels were instrumented with thermocouples. Of these, one contained twelve thermocouples (eight in upper surface, four in lower surface) and the other nine panels contained two each (one in upper surface, one in lower).

### PIV measurement

**Figure 7:**
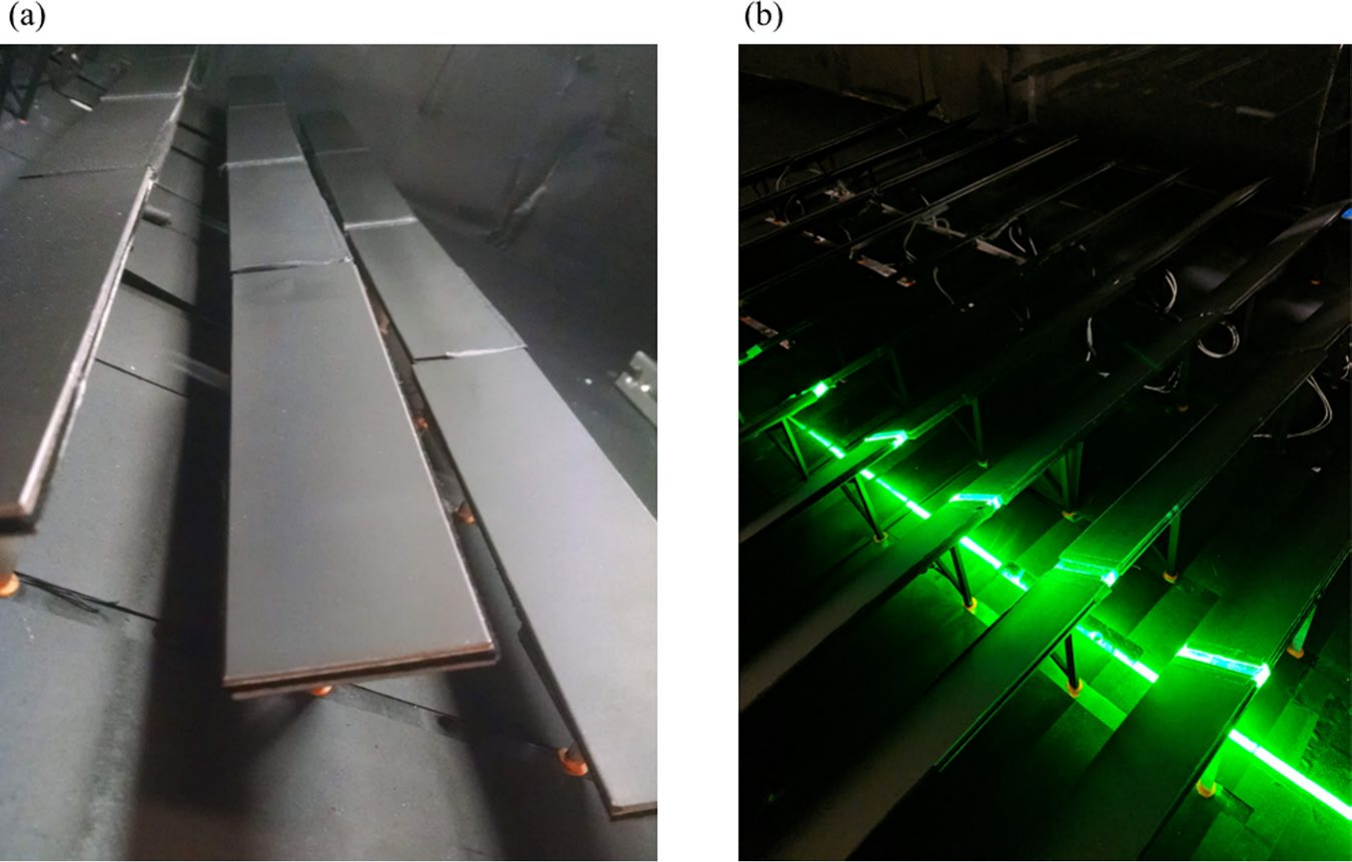
Physical representation of wind tunnel experiments. (**a**) Row eight of the model solar farm and (**b**) Particle image velocimetry of the model solar farm.

Particle image velocimetry was used to capture a 20 *cm* × 20 *cm* flow field, as indicated by the grey box between rows 8 and 9 in Fig. 1. PIV measurements were obtained between the 8th and 9th row, such that the camera could capture the flow over the surface of the 9th panel. A LaVision PIV system was used, with an Nd:Yag (532 *nm*, 1200 *mJ*, 4 *ns* duration) double-pulsed laser and one 4 *MP* ImagerProX charge-coupled device (CCD) cameras. Neutrally buoyant fluid particles (diethylhexl sebacate) were seeded into the flow upstream. In order to capture a plane in the center line of the tunnel, a single camera was aimed between rows, capturing flow in the *x*-*y* plane. The field of view was approximately 0.2 *m* × 0.2 *m* with a vector resolution of 1.5 *mm*. Cameras were calibrated before data was taken on a given plane using a standard two plane measurement plate. The laser plane thickness was approximately 1 *mm*. 3000 PIV image sets were collected for all cases and a convergence test was run calculating ensemble averages of 500, 1000, and 3000 samples. In order to reduce the reflectance for better PIV measurements, a 8 *mm* × 50 *mm* × 6 *mm* piece of transparent acrylic was placed between adjacent panels. This allowed the laser to pass through the surface instead of reflecting and interfering with camera measurements. The vector fields were calculated from raw images using a multi-pass Fast Fourier Transform (FFT) based correlation algorithm. The algorithm used reducing size interrogation windows: twice at 64 × 64 and twice at 32 × 32 pixels with 50% overlap. Each panel was painted matte black on the front and rear to provide a uniform emissivity and prevent reflections during the PIV measurement process. Freestream turbulence intensity was controlled with a grid in the entrance to the test section. See Fig. 7 for a detail of the scaled solar panels.

### Heat transfer theory

The adiabatic convective heat transfer coefficient *h*_*ad*_ was used to quantify the heat transfer to the surrounding air in convection, defined as:

1 $${h}_{ad}=\frac{{q}^{{\prime\prime} }}{{T}_{ref}-\,{T}_{s}}.$$

In this equation *q*″ is the heat flux across either the upper or lower surface and is divided by the thermal potential across that surface *T*_*ref*_ − *T*_*s*_, where *T*_*s*_ is the surface temperature and *T*_*ref*_ is a reference temperature. In this study, the adiabatic surface temperature of the panel was used as the reference temperature, *i*.*e*. the surface temperature when all other panels in the array were heated except for the measured one.
